# Genome wide association study for the identification of genes associated with tail fat deposition in Chinese sheep breeds

**DOI:** 10.1242/bio.054932

**Published:** 2021-05-04

**Authors:** Caiye Zhu, Na Li, Heping Cheng, Youji Ma

**Affiliations:** College of Animal Science and Technology, Gansu Agricultural University, Lanzhou 730070, China

**Keywords:** Genome-wide association studies (GWAS), Post-GWAS, Tail-fat deposition, Sheep, *BMP2*, *PDGFD*

## Abstract

Chinese indigenous sheep can be classified into three types based on tail morphology: fat-tailed, fat-rumped, and thin-tailed sheep, of which the typical breeds are large-tailed Han sheep, Altay sheep, and Tibetan sheep, respectively. To unravel the molecular genetic basis underlying the phenotypic differences among Chinese indigenous sheep with these three different tail types, we used ovine high-density 600K single nucleotide polymorphism (SNP) arrays to detect genome-wide associations, and performed general linear model analysis to identify candidate genes, using genotyping technology to validate the candidate genes. Tail type is an important economic trait in sheep. However, the candidate genes associated with tail type are not known. The objective of this study was to identify SNP markers, genes, and chromosomal regions related to tail traits. We performed a genome-wide association study (GWAS) using data from 40 large-tailed Han sheep, 40 Altay sheep (cases) and 40 Tibetan sheep (controls). A total of 31 significant (*P*<0.05) SNPs associated with tail-type traits were detected. For significant SNPs' loci, we determined their physical location and performed a screening of candidate genes within each region. By combining information from previously reported and annotated biological functional genes, we identified *SPAG17*, *Tbx15*, *VRTN*, *NPC2*, *BMP2* and *PDGFD* as the most promising candidate genes for tail-type traits. Based on the above identified candidate genes for tail-type traits, *BMP2* and *PDGFD* genes were selected to investigate the relationship between SNPs within the tails in the Altay and Tibetan populations. rs119 T>C in exon1 of the *BMP2* gene and one SNP in exon4 (rs69 C>A) of the *PDGFD* gene were detected. rs119 was of the TT genotype in Altay sheep, while it was of the CC genotype in Tibetan sheep. On rs69 of the *PDGFD* gene, Altay sheep presented with the CC genotype; however, Tibetan sheep presented with the AA genotype.

## Abstract (precis)

**Summary:** This is a study on candidate genotyping of sheep with different tail types in China.

## INTRODUCTION

According to the shape of their tails, Chinese indigenous sheep can be divided into three types, namely, fat-tailed, fat-rumped and thin-tailed sheep, of which the typical breeds are large-tailed Han sheep, Altay sheep and Tibetan sheep, respectively.

Large-tailed Han sheep are mainly distributed in the hinterland of the North China Plain, which has a typical temperate continental monsoon climate with obvious changes in the four seasons; cold and dry in winter and hot and rainy in summer. The large-tailed Han belongs to the long fat-tail group, where the fat tail is large, fan-shaped, and hangs down to the hocks. The peach-shaped tail tip is close to the tail groove and is upturned. Altay sheep are mainly distributed in Fuhai and Fuyun counties in the Altay region of Xinjiang Uygur Autonomous Region. This area is dominated by a typical continental climate, with an annual average temperature of 4.0°C, an extreme minimum temperature of −42.7°C, an annual snow cover of 200–250 days, and a snow thickness of 15–20 cm. The fat deposited on the buttocks forms a rounded fat hip, which is wide, straight and rich. There is a shallow groove in the middle of the lower edge of the fat hip, which is divided into two symmetrical halves. Tibetan sheep are native to the Qinghai-Tibet Plateau and are mainly distributed in the Tibet Autonomous Region and Qinghai. The central production area is located at 26°50′∼36°53′ north latitude and 78°25′∼99°06′ east longitude. It is located in the southwestern part of the Qinghai-Tibet Plateau with an average elevation of over 4000 m. The climate is characterized by long periods of sunshine, strong radiation, low temperature, large temperature difference, clear and wet, long night rain, dry winter and spring, high wind pressure, low air pressure and low oxygen content.

Large-tailed Han sheep and Altay sheep store large amounts of fat in their tails. Worldwide, more than 25% of sheep breeds are fat-tailed or fat-rumped (Safdarian et al., 2008). Fat-tailed breeds are an important class of sheep breeds, and were documented for the first time 5000 years ago (Hossein et al., 2012). The fat that is stored in the tail can help during the sheep's migration in winter and with their survival through the cold winter; the fat has additional value to humans because it can provide high-energy food during times of drought and famine (Moradi et al., 2012). Nowadays, customers have increased their meat quality requirements. Key factors for the determination of meat quality are the distribution and content of animal carcass fat. For fat-tailed type sheep, most of the fat is deposited in the tail leading to decreased fat deposits in other parts of the body, which affects meat quality. At the same time, with the construction of herdsmen settlements and forage cultivation technologies continuing to improve, sheep do not need tail fat to provide heat to survive through the cold winter. Moreover, more feed is needed for fat deposition than for meat production, and large tails are not conducive to breeding, therefore, fat deposition in the tail is not so important.

A genome-wide association study (GWAS) is one in which a group of genetic markers that are representative of a phenotype are analyzed for variation within a set of DNA samples (Mccarthy et al., 2008). This method has been used to analyze some economic traits in sheep. For example, Zhang et al. (Zhang et al., 2013) performed a GWAS in purebred sheep for the analysis of 11 growth and meat production traits, and the results indicated that the *MEF2B*, *RFXANK*, *CAMKMT*, *TRHDE*, *RIPK2*, *GRM1*, *POL*, *MBD5*, *UBR2*, *RPL7* and *SMC2* genes were involved in growth and meat production traits in sheep. The mutation of the *TMEM154* gene was identified to be associated with Ovine lentivirus (White et al., 2012). A novel nonsense mutation in the *DMP1* gene was identified by GWAS in Corriedale sheep, which is responsible for inherited rickets (Zhao et al., 2011). Two novel *BMP15* mutations were identified by GWAS, which are responsible for an atypical hyperprolificacy phenotype in sheep (Demars et al., 2013).

Mass spectrometry can be used as a single nucleotide polymorphism (SNP) typing technique based on primer extension (Christian et al., 2004; Wolfgang et al., 2002). The extension primers extend one or several bases at the SNP site to be detected, and then, depending on the fluorescence of the extension product or the molecular mass, they determine its genotype. Time-of-flight mass spectrometry (MALDI-TOF) is an SNP typing method based on the primer extension method combined with matrix-assisted laser desorption/ionization time-of-flight mass spectrometry. Its main advantage lies in the flexible design and high accuracy of the test. The cost performance is best when testing dozens of thousands of samples for dozens of SNPs. This method is suitable for the verification of SNPs found in genome-wide studies and other possible SNPs.

The primary aim of this study was to perform GWAS analysis using an Illumina Ovine SNP600 BeadChip to identify SNPs associated with Chinese indigenous sheep breeds with different tail types at the genome level, and to forecast and explore the major candidate genes associated with fat deposition in sheep tail. The filtered SNP may be used for molecular marker-assisted selection and will guide studies aimed at elucidating some complex traits. As secondary goal was to validate the GWAS results and screen casual genetic variants as genetic markers that are beneficial for the identification of sheep tail type in an independent sheep population, the *BMP2* and *PDGFD* genes were taken to investigate the relationship between SNPs within the tails of Altay and Tibetan sheep.

## RESULTS

### Markers and group information

**Fig. 1.**
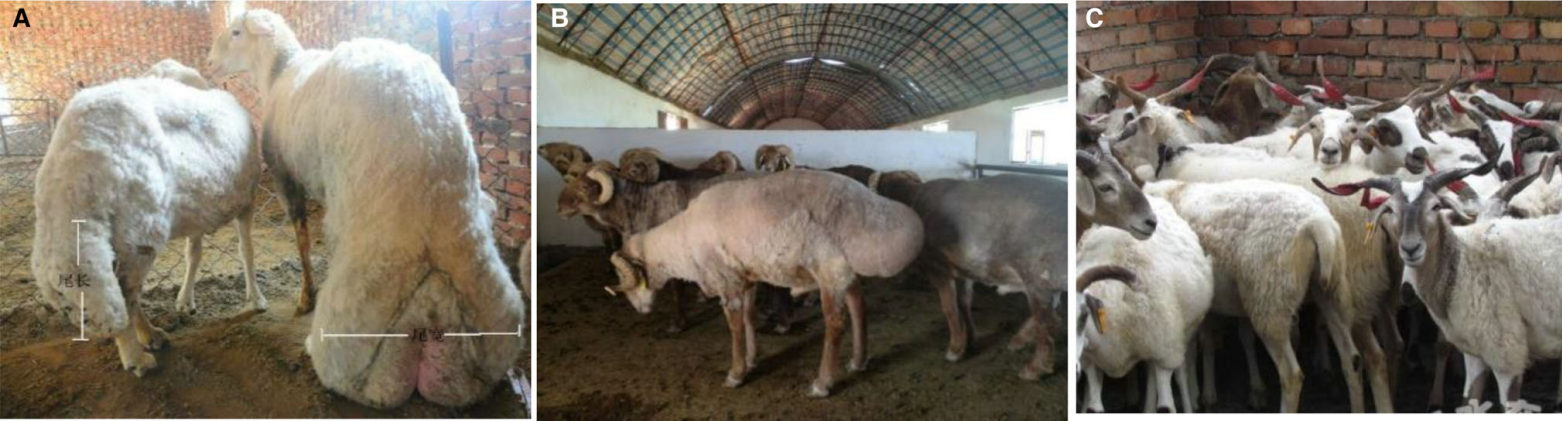
**Test group.** (A) Large-tail Han sheep; (B) Altay sheep; (C) Tibetan sheep.

After quality control, 119 individuals remained (Fig. 1), of which there were 39 large-tailed Han sheep (19 rams and 20 ewes), 40 Altay sheep (20 rams and 20 ewes), and 40 Tibetan sheep (20 rams and 20 ewes) and 538762 SNPs distributed among 26 autosomal.

### PCA analysis

**Fig. 2.**
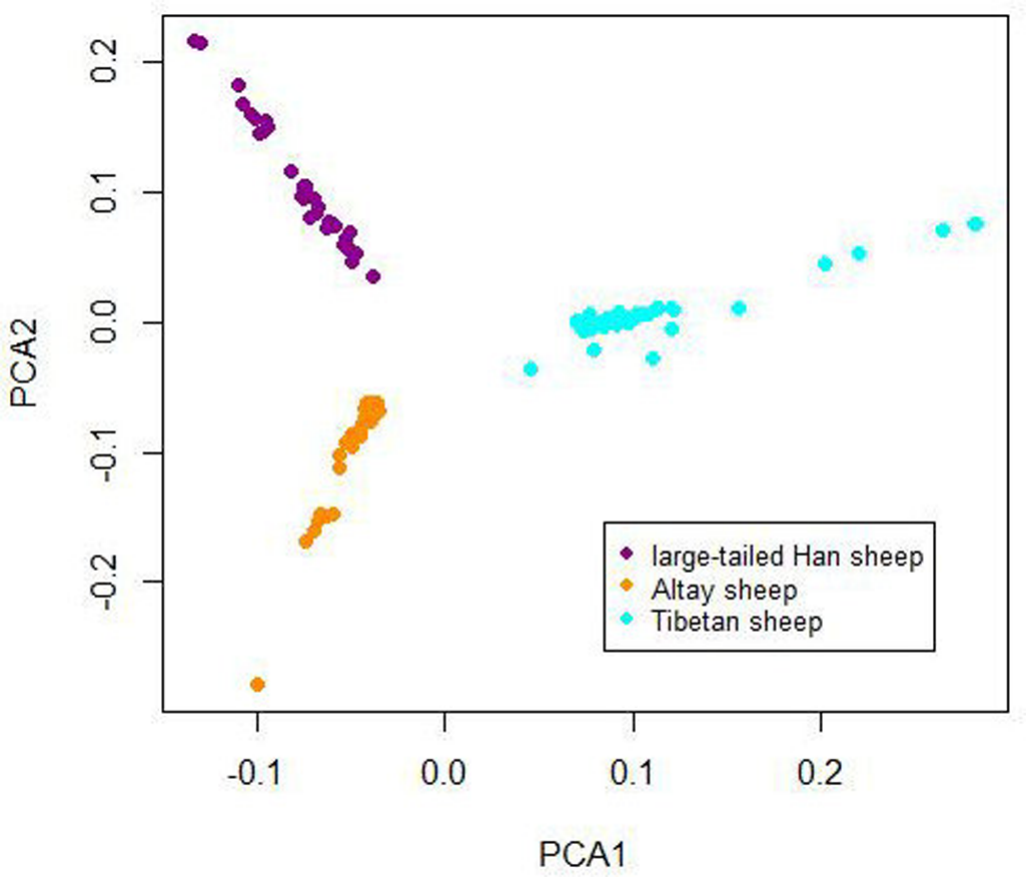
Animals clustered on the basis of principal component (PC) analysis using individual genotypes.

Principal components analysis (PCA) of genome-wide SNPs was performed using R snpStats Software package (Smith and Newton-Cheh, 2009) and Plink software (v1.07; http://pngu.mgh.harvard.edu/purcell/plink). The PCA results showed that the samples from the three breeds were clustered by two principal components: PC1 and PC2. According to PC1, Chinese sheep with different tail types could be divided into two groups consistent with their fat deposition: fat-tailed sheep (PC1<0, large-tailed Han sheep and Altay sheep) and thin-tailed sheep (PC1>0, Tibetan sheep), as shown in Fig. 2.

### The results of GWAS

A case-control design GWAS was performed to research the genetic basis of fat deposition with different tail types. Tibetan sheep were coded as controls and large-tailed Han sheep and Altay sheep as cases. The general linear model (GLM) in Tassel 3.1 was used for whole genome-wide association analysis. We defined the whole-genome significance cut-off as the top 5% (Junwu et al., 2013).

**Fig. 3.**
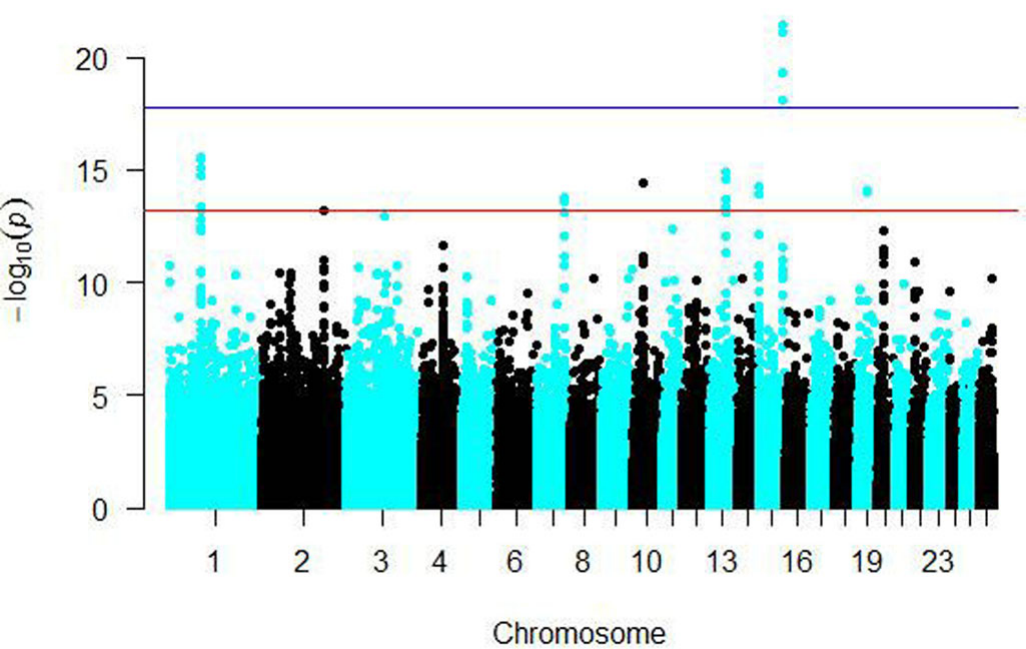
**Results of the GWAS analysis.** The red line represents a significant locus and the blue line represents an extremely significant locus.

In the results as shown in Fig. 3, the different color labels on the X-axis represent different chromosomes, and the Y-axis represents the -log10 (p) of the SNP. The results indicated that a total of 31 genome-wide significant SNPs associated with tail type traits were detected, all of which were distributed on chromosomes 1, 2, 7, 10, 13, 15 or 19. Four strong association signals were observed on chromosome 15.

### Identification of candidate genes with significant SNPs

By comparing genomics and bioinformatics, making full use of the USCS and NCBI databases and the latest sheep Ovis_aries_3.1 genomic information (http://www.ncbi.nlm.nih.gov/assembly/457978) to compare significant SNP loci to confirm their chromosomes and physical locations, the significant SNP was extended approximately 100 kb in the upstream and downstream directions. The results showed that 31 SNPs were significantly (*P*<0.05) associated with tail type at the genome level, distributed on seven different chromosomes, as shown in supplemental (Table S2).

### Enrichment analysis

DAVID v2.1 was used to conduct the gene ontology (GO) and Kyoto Encyclopedia of Gene and Genome (KEGG) pathway enrichment analyses for a more in-depth understanding of the function of these identified genes.

As can be seen from the Table 1, these genes are mainly enriched in biological processes. This GO item mainly involves the regulation of the apoptosis process. KEGG_PATHWAY was involved in melanoma.

**Table 1.**
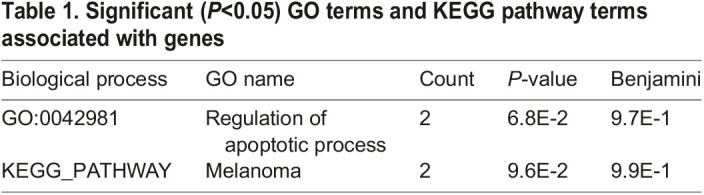
Significant (*P*<0.05) GO terms and KEGG pathway terms associated with genes

### SNP detection and the genotyping of BMP2 and PDGFD

**Fig. 4.**
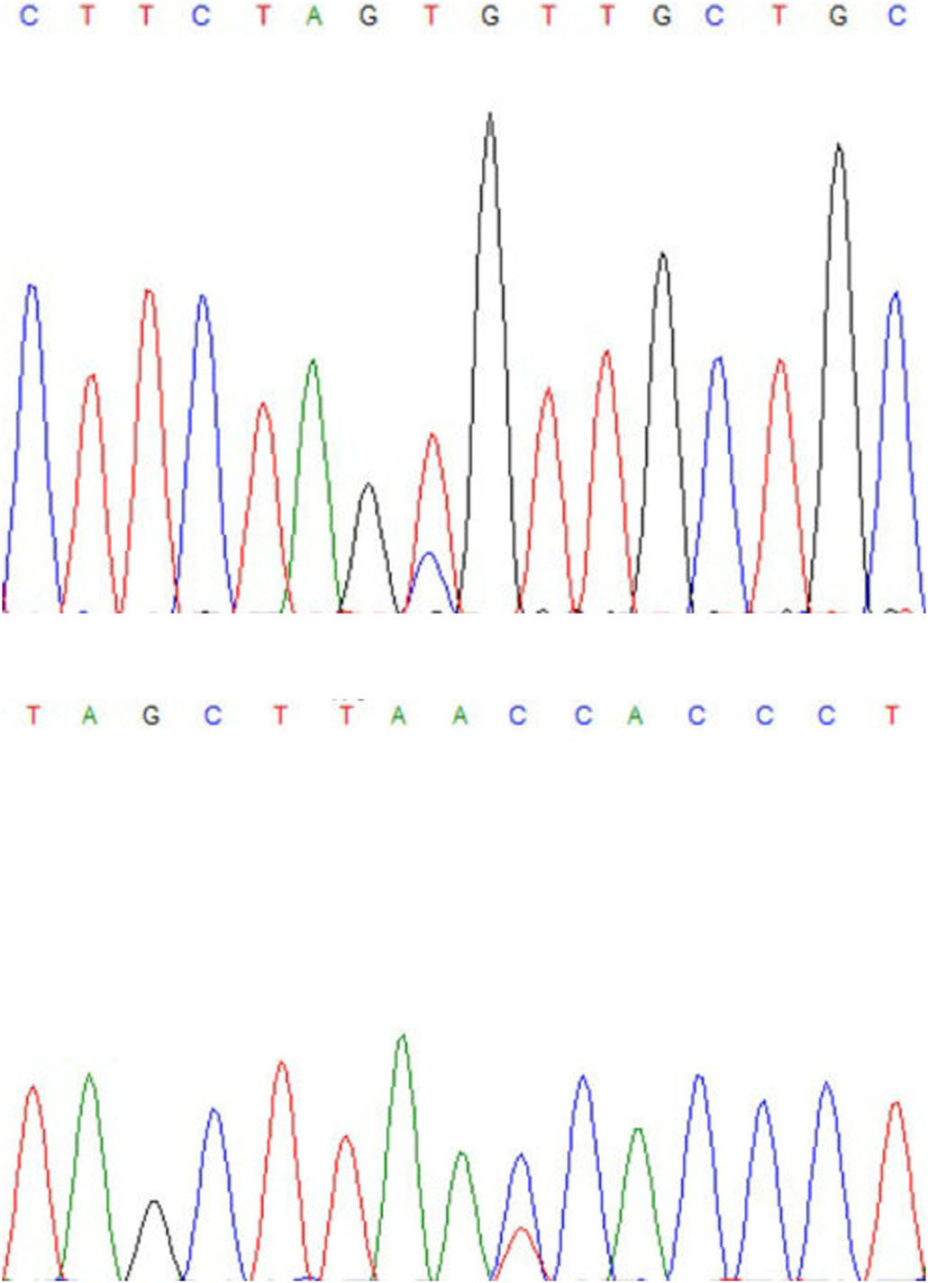
**Sequencing profiles of PCR product from DNA pooling.** (A) rs119 T>C in exon1 of the BMP2 gene; (B) rs69 C>A in exon4 of the PDGFD gene.

DNA pool sequencing showed that one SNP was identified in the first exon of the *BMP2* gene and one SNP in the fourth exon of the *PDGFD* gene. MALDI-TOF assay process used to extend the single site in the, rs119 T>C (Fig. 4A) in exon1 of the *BMP2* gene and rs69 C>A in exon4 (Fig. 4B) of the *PDGFD* gene, respectively, were selected to be genotyped in a new Altay and Tibetan sheep validation group, which included 385 samples.

**Fig. 5.**
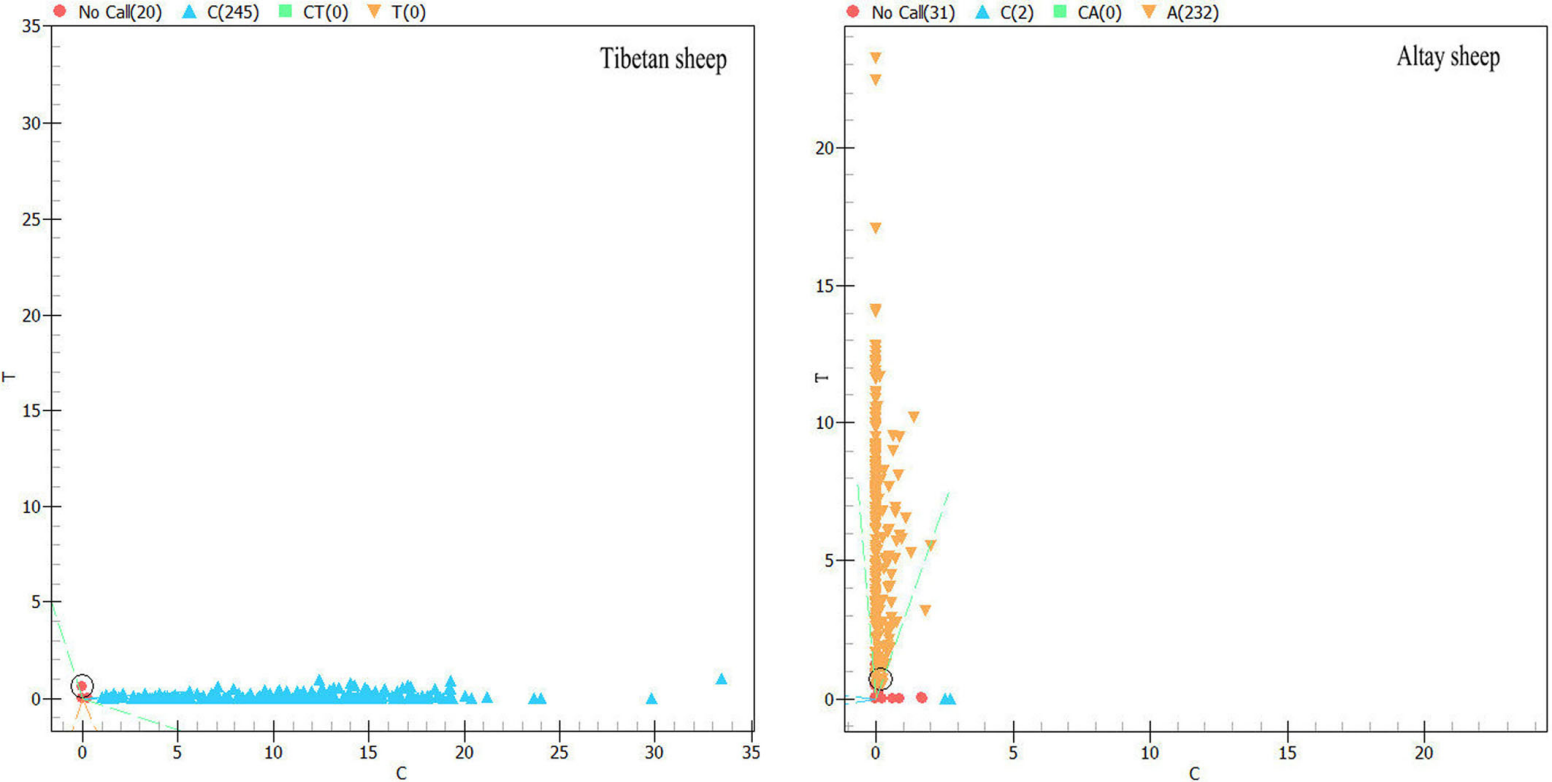
**rs119 locus mass spectrometry results.** ‘C’ means that the genotype of individuals in the blue region was CC; ‘T’ means that the genotype of individuals in the yellow region was TT. Numbers in brackets indicate the number of individuals of the two genotypes.

**Fig. 6.**
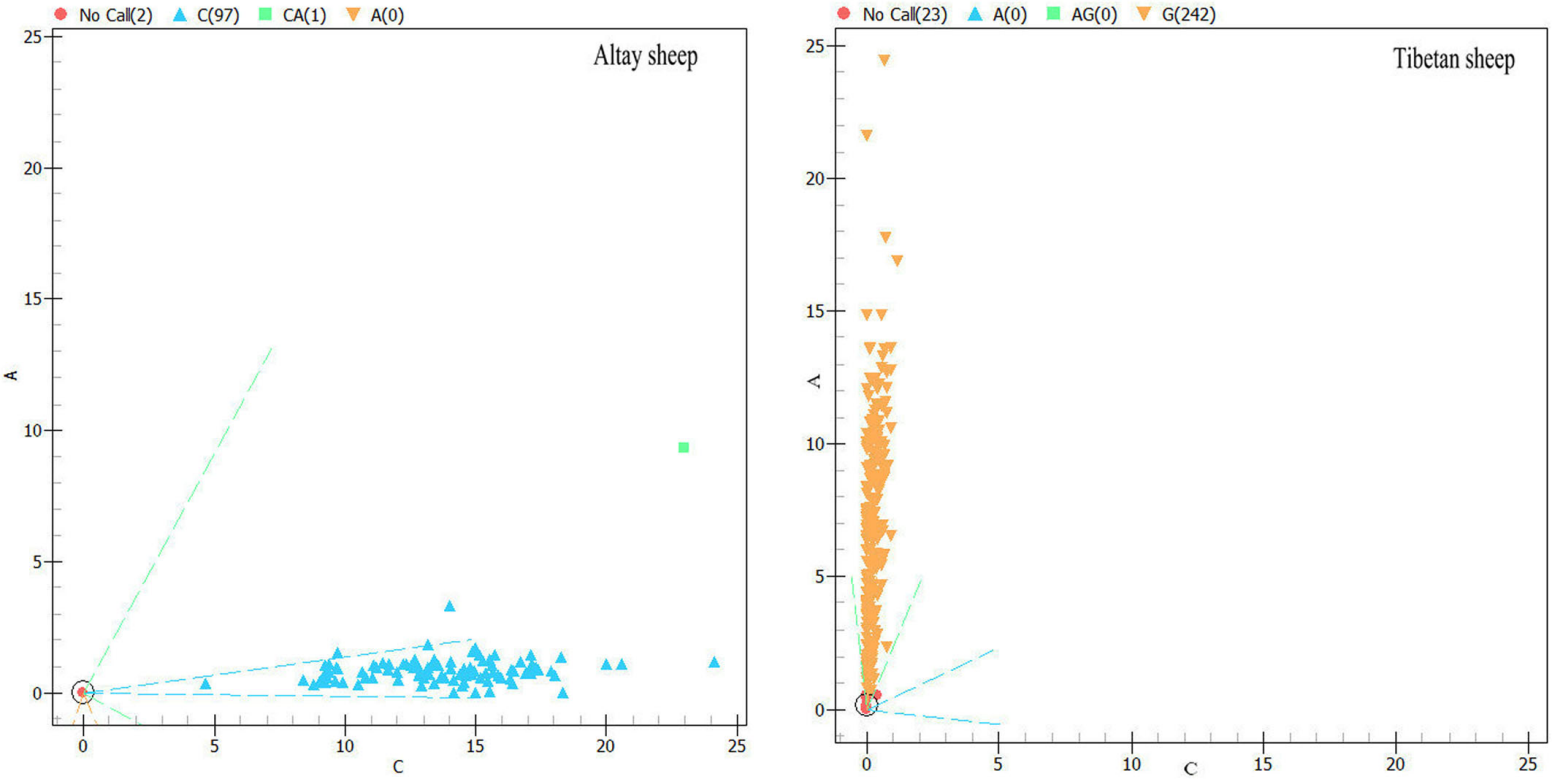
**rs69 locus mass spectrometry results.** ‘C’ means that the genotype of individuals in the blue region was CC; ‘A’ means that the genotype of individuals in the yellow region was AA. Numbers in brackets indicate the number of individuals of the two genotypes.

Mass-array spectrometry was used to genotype the screened SNPs (rs119 and rs69). Each locus has two genotypes (for genotyping results, see Fig. 5 and Fig. 6). rs119 sites are CC, TT, and frequency of the TT genotype is greater than the CC genotype. rs69 is CC, AA, and the frequency of the AA genotype is greater than the CC genotype.

### Association of single nucleotide polymorphism with tail type in Altay and Tibetan sheep

Single SNP association analysis showed that the two genotypes at the rs119 and rs69 loci were significantly different in Altay sheep and Tibetan sheep (*P*<0.05). rs119, which was located on exon1 of the BMP2 gene, was of the TT genotype in Altay sheep, while it was of the CC genotype in Tibetan sheep. On rs69 of the *PDGFD* gene, Altay sheep presented with the CC genotype; however, Tibetan sheep presented with the AA genotype.

## DISCUSSION

In this study, large-tailed Han, Altay and Tibetan sheep, which are national sheep genetic resources of China, were used as the experimental groups. These sheep have different tail types and are distributed in different regions of China. The main producing area of the large-tailed Han sheep is the hinterland of the North China Plains. There are obvious seasonal changes in this region: the winter is cold and dry, and the summer is humid and rainy. Altay sheep live in the Altay Gobi Desert in Xinjiang, where there is an extreme climate, with an average annual temperature of 4°C and a minimum temperature of −42.7°C; the ground is covered by snow for 150–200 days a year. Tibetan sheep live in the Qinghai-Tibet Plateau at an altitude of 3000–5000 meters. Tibetan sheep are relatively strong and tall, and the tail is in the shape of a flat cone. These breeds have different genetic backgrounds, Lanzhou big-tail sheep belong to the Mongolian sheep line, the Altay sheep belong to the Kazakh sheep line, and the Tibetan sheep belong to the Tibetan sheep line. These three breeds represent the three ancient sheep breeds in China, and genes that cause differences in tail types are more easily detected.

In our research, a genome-wide study was performed for three different tail types of sheep breeds; GWAS has been applied to many species but rarely to sheep tail type. TASSEL software has been commonly used in GWAS, and was employed to analyze the associations between SNPs and phenotypes (Bradbury et al., 2007; Zhiwu et al., 2010). In order to make the GWAS research results more reliable, the quality control of individuals and SNP chips can improve the accuracy of data. The permutation test, which calculates the predicted and residual values of the reduced model (contained all terms except markers) then permutes the residuals and adds them to the predicted values; after the quality control of SNPs, the missing genotypes were also filled with BEAGLE software. For statistical methods, we used a generalized linear mixed linear model, which was improved based on the mixed linear model, and its statistical effect was better than other model. From the GWAS results, SNPs that were associated with tail-type traits and were significant at the genome-wide level were located on chromosomes 1, 2, 7, 10, 13, 15, and 19, but mostly on chromosomes 1, 13 and 15. Some of these SNP loci were directly located or close to some genes that have been reported in association with bone, fat metabolism. Zhang et al. (Zhiwu et al., 2010) reported that a directed mutation in *SPAG17* (sperm associated antigen17) may cause bone stagnation in mice, such as shortened hind-limb length, sternal segment fusion, and bone mineralization defects. Gesta et al. (Stephane et al., 2011) reported that *Tbx15* (T-Box Transcription Factor 15) can regulate adipocyte differentiation and mitochondrial respiration, and the *Tbx15* gene plays an important role in limb bone development (Singh et al., 2005). Fan et al. (2013) found that the *VRTN* gene variant was significantly associated with the number of thoracic vertebrae in Chinese and western pigs. The *VRTN* gene was also found in our results, which may affect the tail type of sheep by affecting the number of vertebrae in a sheep's tail. When studying the biological function of the *BMP2* gene in pluripotent stem cells, Ahrens et al. unexpectedly found that under the action of the *BMP2* protein, these cells can not only differentiate into bone cells, but also differentiate into adipocytes (Ahrens et al., 1993). The *BMP2* gene was also detected in our study and may be involved in the formation of fat in sheep tail. *PDGF* (platelet-derived growth factor) is anti-lipogenic and inhibits pre-adipocyte differentiation, but the *PDGF* gene is highly expressed in adipose tissue (Larochelle et al., 2001). Zhu et al. (2016) performed genome-wide detection of CNVs (copy number variations) in Chinese indigenous sheep with different types of tails using ovine high-density 600K SNP arrays. A total of 371, 301, and 66 CNVRs were identified in large-tailed Han sheep, Altay sheep, and Tibetan sheep, respectively. The CNVR regions harbored genes associated with fat deposition and fat synthesis in fat-tailed sheep and fat-rumped sheep. The CNVR regions harbored genes associated with adaptation to the low oxygen environment of the Tibetan plateau in Tibetan sheep. These candidate genes also included *PDGF.* The *PDGFD* gene was identified in this study and is estimated to play an important role in the formation of fat in sheep tail, leading to the formation of different tail types.

To validate the effect of promising SNPs identified by our previous genome-wide association analysis, we selected the *BMP2* and *PDGFD* genes for verification. *BMPs* are important regulators of adipogenesis and may play a role in obesity, and increases in *BMP2* expression may contribute to the partitioning of energy storage into visceral and subcutaneous adipose tissue depots (Guiu-Jurado et al., 2016). In the present study, Altay sheep presented with the TT genotype, while Tibetan sheep presented with the CC genotype. This suggests that this gene of the TT genotype, is likely to be associated with fat deposition in sheep tail. On rs69 of the *PDGFD* gene, Altay sheep presented with the CC genotype; however, Tibetan sheep presented with the AA genotype. This also suggests that this gene of the CC genotype is likely to be associated with fat deposition in sheep tail. In general, phenotypic changes are often caused by functional mutations in genes that control the trait, the mutation site on the gene open frame may affect the changes in the structure of the expressed protein, which in turn will affect the expression of other genes in the signal pathway, and ultimately affect the phenotype, so this mutation can serve as an effective molecular marker for marker-assisted breeding.

## MATERIALS & METHODS

### Ethics statement

All of the animal procedures were performed in strict accordance with the guidelines proposed by the China Council on Animal Care and the Ministry of Agriculture of the People's Republic of China. All of the animal experiments were approved by the Gansu Agricultural University (Lanzhou, China), approval no. GSAU-AEW-2017-0003.

### DNA sample collection

In our study, 120 animals, were from Liaocheng in Shandong Province, including 40 large-tailed Han (20 rams and 20 ewes), Altay in Xinjiang Province, including 40 Altay sheep (20 rams and 20 ewes), and Tianzhu in Gansu Province, including 40 Tibetan sheep (20 rams and 20 ewes), respectively. These samples were collected from research station, the flock size is greater than 1000. All of the experimental animals were randomly selected, then blood was collected from the jugular vein, 8 ml blood was collected from each sheep, and stored at −20°C after slight shaking.

A TIANamp Blood DNA Kit (Tiangen Biotech Co. Ltd., Beijing, China) was used to extract Genomic DNA from samples of whole blood. A NanoVue Spectrophotometer producer was used to assess the purity and concentration of genomic DNA.

### Genotyping and quality control

The Illumina Ovine SNP 600 BeadChip (Illumina Inc., CA, USA), which contained 604,715 SNPs spanning the ovine genome, was used to genotype the genomic DNA of each animal, with an average distance of 4.28 Kb between two SNPs.

PLINK software (v1.07; http://pngu.mgh.harvard.edu/purcell/plink/) was used to control the quality of the genotype data. Stringent quality control criteria were applied to increase the accuracy of GWAS inference: (1) sample call rate >95% and SNP call frequency >90%, (2) minor allele frequency (MAF) >3%, (3) Hardy–Weinberg equilibrium test, *P*<10–6. After quality control, 58762 SNP sites remain.

At the same time as the abovementioned genotyping, when performing genotyping, some of the SNP data obtained in the experiment were not successfully typed. Therefore, Beagle (v.4.0) (Browning and Browning, 2009) software was used to fill in the missing genotype data. The principle is to construct a haplotype using linkage disequilibrium between markers, and approximate the expected value of the missing genotype. Beagle fill command was as follows: java -Xmx1000 m -jar beagle.jar unphased=beagle-chr.bgl missing=0 out=example.

### Phenotypic trait determination

The phenotypic traits determined by this experiment included: tail length, tail width and tail circumference. Tail length refers to the distance in the fat-tailed sheep from the leading edge of the first caudal vertebra to the tail tip. Tail width refers to the straight line distance at the widest point of the tail. The tail circumference is the length of one circle around the tail tip. The tail length, width and circumference of Lanzhou big-tailed sheep are 38, 32, and 106 cm, respectively. The tail length, width and circumference of Altay sheep are 22, 36, and 98 cm, respectively. The tail length, width and circumference of Tibetan sheep are 15, 4, and 7 cm, respectively.

### Genome-wide association analysis

In this study, genome-wide association analysis was performed using a case-control analysis method to compare the frequency of genotypes of large-tailed and thin-tailed individuals (alleles) at each marker locus in the genome. In order to correct the possible false-positive effects of genome-wide association analysis by genetic relationship and population stratification, PCA was conducted using snpStats (v.1.4.0) in R (v.4.0) (http://cran.r-project.org), a more general analysis model – the GLM (Zhang et al., 2009) – was used to perform genome-wide associations using TASSEL (v.3.0) (Bradbury et al., 2007) software. The permutation test was run using the method suggested by Anderson and Ter Braak (Anderson and Braak, 2003). The mathematical expression of this model is:
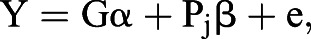
where Y is a phenotypic observation vector, α and β are correlation matrices, G is the effect vector of SNP, P_j_ is the first and second principal component matrix in a generalized linear model, and e is the random residual effect vector.

### Gene detection and functional analysis

By comparing genomics and bioinformatics, making full use of the UCSC (http://genome.ucsc.edu/cgi-bin/hgGateway?redirect=manual&source=genome.ucsc.edu) and NCBI databases and the latest sheep Ovis_aries_3.1 genomic information (http://www.ncbi.nlm.n.gov/assemblv/457978/), significant SNP loci were aligned to confirm their chromosomes and physical locations, and gene function annotations were performed in the 100 Kb region upstream and downstream of the SNP. The Database for Annotation, Visualization and Integrated Discovery (DAVID) (Huang Da Wei and Lempicki Richard, 2009; Lempicki, 2009) (http://david.abcc.ncifcrf.gov/) was used to perform the GO enrichment analysis and KEGG pathway analysis. To better understand the functions of the genes within the 100 Kb region upstream and downstream of the SNP, the Ovis aries Ensembl gene IDs were converted into human orthologue Ensembl gene IDs using BioMart (http://www.biomart.org/), since the annotation of the sheep genome is limited.

### SNP detection and genotyping of candidate genes of BMP2 and PDGFD

In this experiment, 385 blood samples (200 are Altay sheep and 185 are Tibetan sheep) were collected from Altay sheep in Altay, Xinjiang, and from Tibetan sheep. Genomic DNA extraction of the collected blood samples was performed using the TIANamp Blood DNA Kit, the detection of DNA sample concentrations using NanoDrop (Thermo NanoDrop 2000) and the detection of DNA quality by 0.8% agarose gel electrophoresis. 30 DNA samples were randomly selected and evenly diluted to 50 ng/μl, and then 2 μl of each sample was taken and evenly mixed to construct the DNA pool.

### SNP detection and genotyping

PRIMER 3 (v.0.4.0.) (http://bioinfo.ut.ee/primer3-0.4.0/) online design software was used to design primers for all exons of the gene. Primer information is shown in the Supplementary file (Table S1). Primers were synthesized by Cybrex Biotech Co., Ltd. The total PCR reaction system was 20 μl:1 μl of 5′ primers, (10 pmol/μL), 1 μl of 3′ primers (10 pmol/μL), 10 μl for the 2×Taq PCR Master Mix (Taq DNA polymerase (0.05 U/µl), reaction buffer, 4 mM MgCl2, 0.4 mM of each dNTP and 4×1.25 ml Nuclease-free water. Producer of Taq pol and dNTPs Tiangen Biotech Co. Ltd., Beijing, China), 2 μl for the DNA template, and 6 μl for the ddH_2_O. PCR reaction program: 94°C pre-denaturation 5 min; 94°C denaturation 30 s, annealing temperature 30 s, 72°C extension 30 s, 35 cycles; 72°C extension 10 min; 4°C preservation. The PCR product was detected by 0.8% agarose gel electrophoresis, and the target band was sent to Bio Miao Biological Technology (Beijing) Co., Ltd. for sequencing. According to the sequencing results, sequence comparisons using DNAMAN 5.2.10 software (Lynnon BioSoft, Quebec, ONT, Canada) and Chromas (v.2.0) software were performed to screen for SNP sites.

Using Mass-Array mass spectrometry technology (Mass ARRAY; Sequenom Inc., San Diego, CA, USA), two SNPs were then genotyped in 200 Altay and 185 Tibetan sheep for genotyping. According to the sequence information of the SNP site, use the primer design software Assaydesign of Sequenom Company 3.1 to design the PCR reaction and single base extension primers and synthesize them. After the sample resin is purified, use Mass ARAY Nanod Ispenser points the purified product and transfers it to the Spectro CHIP (Sequen-om) chip, and uses matrix-assisted laser desorption/ionization time-of-flight mass spectrometry for analysis. The detection results are typed using TYPER 4.0 software and the results are output.

### Data processing and statistical analysis

SPSS 22.0 software and a case-control analysis method were used to compare the frequency of genotypes of large-tailed and thin-tailed individuals (alleles), at each marker locus in the genome. The statistical model is as follows:
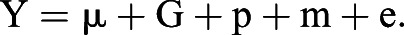
In the formula, Y represents the measured value of the trait, μ represents the population mean, G is the *BMP2* and *PDGFD* genotype effect, p is the field effect, m is the gender effect, and e is the random residual.

## Conclusions

Chinese indigenous sheep can be classified into three types based on tail morphology. Some candidate genes were identified by genome-wide association analysis and genotyping technology, and it was discovered that on exon1 of the *BMP2* gene, Altay sheep presented with the TT genotype, while Tibetan sheep presented with the CC genotype. On exon4 of the *PDGFD* gene, Altay sheep presented with the CC genotype; however, Tibetan sheep presented with the AA genotype. These genes can be molecular markers for the selection of sheep tail type.

## Supplementary Material

Supplementary information
