# Supplementary material for: Genome wide association study for the identification of genes associated with tail fat deposition in Chinese sheep breeds
Source: Biol Open. 2021 May 4;10(5):bio054932. doi: 10.1242/bio.054932 (PMC8186729; doi:10.1242/bio.054932)
Supplement: Supplementary information [file biolopen-10-054932-s1.pdf]

Table S1 Information of primer sequences for scanning SNPs within sheep BMP2 and PDGFD gene

BMP2-exons1-F	CTTAAGTATCCCCGCGAGTG
BMP2-exons1-R	ACACGTTTCGCAGTCTAGGG
PDGFD-exons4-F	GGTACCAGGTATGATTTTGTAG
PDGFD-exons4-R	CTTCATTTAACCAGTGTCTGCT

Table S2. Functional annotation for chromosome-wide significant SNPs associated with TW

SNP name	chr	position(bp)	P value	Gene name
oar3_OAR1_94912049	1	94912049	8.04E-16	
oar3_OAR1_94931158	1	94931158	1.75E-15	
oar3_OAR1_94940531	1	94940531	1.75E-15	
oar3_OAR1_94977426	1	94977426	3.47E-16	*SPAG17*
oar3_OAR1_95037331	1	95037331	6.03E-14	
oar3_OAR1_95069631	1	95069631	4.06E-14	
oar3_OAR1_95596363	1	95596363	2.64E-16	
oar3_OAR1_95597675	1	95597675	2.64E-16	
oar3_OAR1_95600207	1	95600207	2.64E-16	*TBX15, WARS2*
oar3_OAR2_183136068	2	183136068	6.28E-14	*EN1, MARCO*
s55494.1	7	82545552	1.75E-14	ALDH6A1
oar3_OAR7_82603868	7	82603868	2.30E-14	
oar3_OAR7_82623517	7	82623517	7.33E-14	*LIN52, VSX2, ABCD4, VRTN, TMEM90A, NPC2,* ISCA2, LTBP2
oar3_OAR10_29373640	10	29373640	3.50E-15	*FRY, EEF1A1, RXFP2*
oar3_OAR13_48651711	13	48651711	7.82E-14	*BMP2*
oar3_OAR13_48882107	13	48882107	4.01E-14	
oar3_OAR13_48890947	13	48890947	7.18E-14	
oar3_OAR13_48918153	13	48918153	2.40E-15	
oar3_OAR13_48920964	13	48920964	2.40E-15	
oar3_OAR13_48927268	13	48927268	2.40E-15	*PPP1CC*
oar3_OAR13_49290888	13	49290888	1.12E-15	
oar3_OAR13_49330252	13	49330252	2.18E-14	
oar3_OAR13_49354226	13	49354226	2.18E-14	*MUTED*
oar3_OAR15_3870680	15	3870680	5.65E-15	
oar3_OAR15_3871017	15	3871017	1.14E-14	*PDGFD, DDI1,*

Biology Open • Supplementary information

oar3_OAR15_72542670	15	72542670	3.63E-22	
oar3_OAR15_72543368	15	72543368	7.62E-22	
oar3_OAR15_72547431	15	72547431	4.50E-20	
oar3_OAR15_72549351	15	72549351	7.83E-19	*ACCSL, EXT2, ALX4*
oar3_OAR19_31653185	19	31653185	7.98E-15	
oar3_OAR19_31657197	19	31657197	9.90E-15	*MITF*
